# Künstliche Intelligenz zur Unterstützung der Telemedizin am Beispiel Afrikas

**DOI:** 10.1007/s00105-020-04664-6

**Published:** 2020-08-06

**Authors:** C. Greis, L. V. Maul, C. Hsu, V. Djamei, P. Schmid-Grendelmeier, A. A. Navarini

**Affiliations:** 1 Klinik für Dermatologie, Universitätsspital Basel, Basel, Schweiz; 2 Klinik für Dermatologie, Universitätsspital Zürich, Gloriastr. 31, 8091 Zürich, Schweiz

**Keywords:** Teledermatologie, Digitalisierung, Bilderkennung, Automatisierte Algorithmen, Smartphone-Bilder, Teledermatology, Digitization, Image recognition, Automated algorithms, Smartphone images

## Abstract

Telemedizin findet seit Jahrzehnten Anwendung im Alltag von Dermatologen. Insbesondere in afrikanischen Ländern mit begrenzter medizinischer Versorgung, zu überbrückenden geografischen Distanzen und einem zwischenzeitlich relativ gut ausgebauten Telekommunikationssektor liegen die Vorteile auf der Hand. Nationale und internationale Arbeitsgruppen unterstützen den Aufbau von teledermatologischen Projekten und bedienen sich in den letzten Jahren zunehmend KI(künstliche Intelligenz)-gestützter Technologien, um Ärzte vor Ort zu unterstützen. Vor diesem Hintergrund stellen ethnische Variationen eine besondere Herausforderung in der Entwicklung automatisierter Algorithmen dar. Um die Genauigkeit der Systeme weiter zu verbessern und globalisieren zu können, ist es wichtig, die Zahl der verfügbaren klinischen Daten zu erhöhen. Dies kann nur mit der aktiven Beteiligung der lokalen Gesundheitsversorger sowie der dermatologischen Gemeinschaft gelingen und muss stets im Interesse des einzelnen Patienten erfolgen.

## Abstract (en)

Telemedicine has been used in the daily routine of dermatologists for decades. The potential advantages are especially obvious in African countries having limited medical care, long geographical distances, and a meanwhile relatively well-developed telecommunication sector. National and international working groups support the establishment of teledermatological projects and in recent years have increasingly been using artificial intelligence (AI)-based technologies to support the local physicians. Ethnic variations represent a challenge in the development of automated algorithms. To further improve the accuracy of the systems and to be able to globalize, it is important to increase the amount of available clinical data. This can only be achieved with the active participation of local health care providers as well as the dermatological community and must always be in the interest of the individual patient.

## Teledermatologie in Afrika – in Zeiten von COVID-19 und darüber hinaus

Nicht zuletzt während der COVID-19-Pandemie hat die Teledermatologie zunehmende Akzeptanz in der Bevölkerung gefunden – seitens Ärzten und Patienten. Weltweit entfallen ca. 30 % aller telemedizinischen Anwendungen auf die Dermatologie mit einer jährlichen Wachstumsrate von 8,7 % [1].

Insbesondere in Entwicklungsländern mit einer begrenzten Anzahl qualifizierter Dermatologen hat die Teledermatologie ein besonderes Potenzial, zur Verbesserung der medizinischen Versorgung von Hautkrankheiten beizutragen.

**Figure 1:**
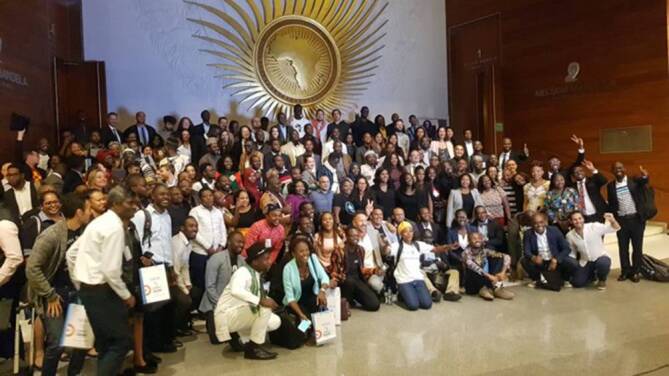

Bereits vor 20 Jahren sammelten Schmid-Grendelmeier et al. erste internationale, teledermatologische Erfahrungen in Afrika [2]. Seither wurden diverse Projekte verteilt über den gesamten Kontinent z. B. in der Subsahara [3], Burkina Faso [4] oder Ghana [5] lanciert, um lokale Ärzte, Dermatologen und Mitarbeiter in Gesundheitseinrichtungen zu unterstützen. Einige Vorhaben finden im Rahmen von interkontinentalen Kooperationen statt, andere werden durch Förderungen im lokalen Ökosystem (vgl. Abb. 1 und 2) vorangetrieben.

**Figure 2:**
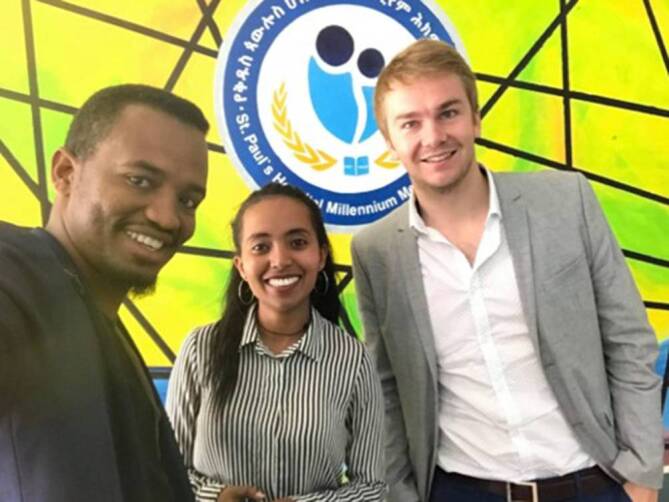

Anders als in Europa, wo mittels öffentlicher Verkehrsmittel selbst finanziell sehr schwache Patienten ein tertiäres Zentrum erreichen können, ist die Distanz in Afrika weiterhin in vielen Regionen ein großes Problem. Die Patienten müssen teils Hunderte von Kilometern hinter sich bringen, um die Klinik zu erreichen. Dermatologische Krankheiten werden von vielen Patienten immer noch als weniger relevant im Vergleich zu internistischen Krankheiten wahrgenommen, weshalb aufwendige Reisen oftmals nicht erfolgen und die Haut unbehandelt bleibt. Abgesehen von der Nichtverfügbarkeit von Einrichtungen und schlechter Kommunikation haben die meisten Afrikaner ein unterschiedliches Verständnis von Krankheiten, die häufig das Gesundheitssystem betreffen [6]. Gerade in einer Disziplin wie der Dermatologie, wo beispielsweise bei Infektionen, Tumoren oder Wunden eine sehr rasche Evaluation des Krankheitsbildes vorkommt, sind zudem Nachsorgeuntersuchungen bei solchen Distanzen für die Patienten teils kaum machbar. Deswegen ist eine telemedizinische Konsultation mittels Fotos und Anamnesetext sehr attraktiv. Nur der kleinste Teil der Bevölkerung ist digital herausgefordert, die notwendigen Gesundheitsdaten zu sammeln und einzusenden. Insbesondere die jüngeren Generationen in Afrika haben genau gleich wie in Europa eine intensive Exposition zu digitalen Kommunikationsmitteln und nutzen diese nicht selten täglich.

Nicht zuletzt die raschen Fortschritte im Telekommunikationssektor, insbesondere die Verbreitung von Smartphones sowie die Einführung von 4G (LTE), bieten in diesen Ländern zunehmende Möglichkeiten für die Teledermatologie. So liegt inzwischen die Penetranz von Smartphone-Abonnements in Afrika bei durchschnittlich 45 %. Ab 2022 wird ein erkennbares Volumen von 5G-Abonnements erwartet, das bis 2025 3 % erreichen wird. Zu den treibenden Faktoren für das Wachstum mobiler Breitbandabonnements gehören eine junge, wachsende Bevölkerung mit zunehmenden digitalen Fähigkeiten und erschwinglichere Smartphones [7].

Grundsätzlich unterscheidet man 2 verschiedene Verfahren in der Teledermatologie: Bei der Life-Interaction-Methode erfolgt eine synchrone Kommunikation zwischen Arzt und Patient mittels Videokonferenz. Bei der Store-and-Forward-Methode werden Bilder zusammen mit der klinischen Anamnese des Patienten gespeichert und zeitlich verzögert vom Arzt bearbeitet. Die Life-Interaction-Teledermatologie kann durch die direktere Kommunikation oft klarere klinische Informationen über den Patienten liefern. Store-and-Forward-Teledermatologie ist in der Regel kostengünstiger für den Anbieter der Gesundheitsdienstleistung und bietet darüber hinaus die Grundlage für Algorithmen-basierte Analysen der Daten bis hin zur künstlichen Intelligenz. Sie eignet sich unseres Erachtens weitaus besser für die standardisierte Behandlung einer größeren Zahl von Patienten.

Die Store-and-Forward-Teledermatologie eignet sich besser für die Behandlung einer größeren Patientenzahl

Anders als in den industrialisierten Ländern, in denen die Teledermatologie nur langsam Anklang findet und dies primär bei jüngeren, technisch interessierten und zeitlich schwierig abkömmlichen Patienten, gibt es für die Teledermatologie in Afrika eine andere Ausgangslage. Es existieren Millionen von Personen, die normalerweise aufgrund multifaktorieller Ursachen gar keine Möglichkeit haben, einen Dermatologen zu konsultieren. Die Teledermatologie stellt für diesen Teil der Bevölkerung nun ein neues Portal für eine Behandlung dar, die normalerweise nicht erreichbar gewesen ist.

## Bessere Anwendbarkeit von künstlicher Intelligenz dank Teledermatologie

Künstliche Intelligenz (KI) hat sich zu einem relevanten Forschungsthema in der Medizin entwickelt und wird zunehmend in der Dermatologie angewandt. Die meisten KI-Anwendungen beschäftigten sich mit der Unterscheidung zwischen gutartigen und bösartigen Hautläsionen [8, 9], andere mit entzündlichen Hautkrankheiten [10], der Allergologie [11] oder Dermatopathologie [12, 13]. Obwohl die Zahl der Studien zunimmt, gibt es interessanterweise relativ wenige Arbeiten, bei denen Dermatologen an der Konzeption, Gestaltung und Interpretation der Studien maßgeblich beteiligt sind. Die meisten Studien werden von wissenschaftlich tätigen Ingenieuren geleitet [14].

Frühere Bemühungen, KI in der alltäglichen Dermatologie zu etablieren, haben nur begrenzte Erfolge gezeigt, da es bislang an konkreten Anwendungsfällen in der klinischen Praxis mangelte und die Algorithmen anhand von standardisierten Bildern entwickelt wurden. Der jüngste Lernerfolg bei der automatisierten Diagnose von Hautläsionen anhand von klinischen, teils niedrig auflösenden Smartphone-Bildern ermutigt, dass diese Technologien nun bereit sind, in der Praxis getestet bzw. der Praxis zugänglich gemacht zu werden. Somit wird die KI in der Telemedizin zu einer erreichbaren Realität, um die Möglichkeiten der dermatologischen Versorgung zu erweitern und die Kosten weiter zu senken. Zum Beispiel könnte KI als klinisch-diagnostisches Unterstützungsinstrument für Teledermatologen dienen oder mehrdeutige bzw. maligne Pathologien direkt an den nächsten Arzt weiterleiten.

## Dermatologen sehen Chance im Einsatz von künstlicher Intelligenz

In einer kürzlich durchgeführten internationalen Online-Umfrage unter 1271 Dermatologen wussten 85,1 % der Befragten, dass die KI ein aufkommendes Thema in ihrem Fachgebiet ist. Darüber hinaus stimmten 77,3 % der Befragten zu, dass die KI die dermatologische Versorgung verbessern wird, nur 5,5 % der teilnehmenden Dermatologen äußerten die Befürchtung, dass KI-Anwendungen Dermatologen in naher Zukunft ersetzen würden [15]. Auch die WHO (World Health Organization) sieht ein großes Potenzial in der KI bei der Gesundheitsversorgung in diesen Regionen [16].

## Ethnische Variationen als Herausforderung in der Entwicklung von Algorithmen

Viele bisher entwickelte Algorithmen wurden basierend auf klinischen Bildern von Kaukasiern mit Hauttyp I–III nach Fitzpatrick entwickelt. Die herkömmliche klinische Erfahrung zeigt, dass sich Pathologien abhängig vom Hauttyp in ihren Effloreszenzen (insbesondere Rötung, Schuppung, Lichenifikation) unterschiedlich präsentieren können. Auch unterscheiden sich dermatologische Krankheitsbilder, wie z. B. die Erscheinungsformen der atopischen Dermatitis, weltweit. In Afrika weisen Patienten mit atopischer Dermatitis vermehrt follikuläre und lichenoide Läsionen auf, wohingegen in Asien eher Lichen simplex chronicus, Lichenifikationen und Prurigo-nodularis-Formen vorkommen und in Indien und im Iran weniger häufig eine flexurale Beteiligung vorliegt [17]. Anlässlich eines Treffens mit Ärzten aus Afrika südlich der Sahara wurde die atopische Dermatitis als eine der mit Abstand häufigsten entzündlichen Dermatosen in dieser Region identifiziert und digitale Methoden zu deren Erkennung und Beratung als sehr wesentlich postuliert [18].

In einem Pilotversuch, in dem ein KI-Modell in einer asiatischen Population trainiert und anschließend in einer kaukasischen Population validiert wurde, zeigte sich eine Fehleranfälligkeit [19]. Um die Genauigkeit der Systeme diesbezüglich weiter zu verbessern und globalisieren zu können, ist es wichtig, die Zahl der verfügbaren klinischen Bilder von Patienten unterschiedlichen Alters und unterschiedlicher ethnischer Herkunft zu erhöhen.

Einfache Probleme müssen möglichst automatisiert behandelt werden

**Figure 3:**
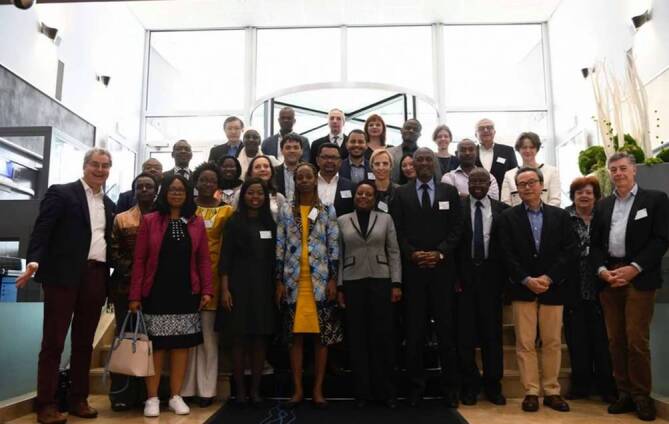

Vor diesem Hintergrund haben sich weltweit mehrere Forschungsgruppen dieses Themas angenommen. Das Africa Teledermatology Project, unterstützt durch die Kommission für Entwicklungsfragen der Österreichischen Akademie der Wissenschaften sowie die American Academy of Dermatology, baut seit Jahren eine entsprechende Datenbank zu Zwecken der Lehre und Forschung auf. Auch das Universitätsspital Basel hat mit weiteren international anerkannten Universitätskrankenhäusern das Projekt PASSION ins Leben gerufen mit dem Ziel der Integration von KI in der pädiatrischen Dermatologie in Entwicklungsländern (Abb. 3). Wir haben hier unsere Erfahrung in der Teledermatologie sowie das Arbeiten in Tansania und anderen Ländern zusammengelegt, um Lösungen zu finden, Patienten telemedizinisch an der Haut zu behandeln. Da wir, wie oben skizziert, von vielen Personen ausgehen, die in Afrika Teledermatologie wahrnehmen möchten, benötigen wir von Anfang an Lösungen, die auch für eine große Zahl an Patienten tragfähig sind. Diese müssen sich darauf konzentrieren, die einfachen Probleme möglichst automatisiert behandeln zu können, damit die Expertise der wenigen menschlichen Dermatologen für die Evaluation und Behandlung komplexer Probleme reserviert bleiben kann.

So haben wir in dem Projekt PASSION das Ziel, 5 visuell einfach identifizierbare Dermatosen bei Kindern mittels KI zu erkennen und nach einem standardisierten Ablauf zu behandeln. Die Güte dieser Behandlungsstrategie wird in einer Studie zu Anfang direkt einer persönlichen Konsultation beim Dermatologen sowie einer Telekonsultation beim menschlichen Dermatologen gegenübergestellt. Sollte sich dieses Vorgehen als genügend erweisen, würde das Projekt von einer Studie zu einem für die gesamte Bevölkerung offenen medizinischen Angebot weiterentwickelt werden. Dies wird nicht zur Abschaffung der persönlichen dermatologischen Konsultation führen, da nicht alle Dermatosen mittels Teledermatologie identifiziert und behandelt werden können Wir werden über mehrere Jahre eine mindestens 10- bis 40 %ige Rate an Fällen haben, bei denen die Diagnose nicht sicher gestellt werden kann. Dies zeigt sich auch anhand einer Applikation aus den Niederlanden, die sich im Direct-to-consumer-Bereich der Evaluation von Nävuszellnävi bewegt. In diesem visuell besonders schwierigen Gebiet der Hauttumoren hatte die Applikation eine hohe Fehlerquote [20]. Deshalb braucht es in vielen Fällen weiterhin, und insbesondere bei visuell schwierig beurteilbaren Krankheiten, die persönliche Interaktion mit dem Dermatologen. Der PASSION-Ansatz zielt daher in die Richtung einer Automatisierung von einfachen Situationen und damit Entlastung der wenigen Dermatologen, was wir als durchaus erreichbar einschätzen.

## Grenzen und Fallstricke der Telemedizin – Handeln im Interesse des Patienten

Das Interesse des einzelnen Patienten sollte stets im Vordergrund stehen. Bei jeder telemedizinischen Konsultation sollten die Grenzen der Telemedizin im Vergleich zur herkömmlichen persönlichen Konsultation abgewogen werden. Nicht zuletzt vor dem Hintergrund einer möglichen Haftungsfrage sollte insbesondere das Hinzuziehen von KI nur ein Orientierungsinstrument und kein absolutes Diagnoseinstrument darstellen.

Beim Angebot entsprechender Technologien wird dem Patienten ein größeres Maß an Eigenverantwortung im Vergleich zu herkömmlichen Konsultationen übertragen, wobei soziodemografische Faktoren wie jüngeres Alter und höherer Bildungsgrad sich ungleich auf den Therapieerfolg auswirken können. Notwendige Voraussetzungen wie das Vorhandensein von Internet, Hardware und nicht zuletzt technischen Fähigkeiten des Einzelnen dürfen nicht als selbstverständlich angenommen und Technologien alternativlos angeboten werden.

## Fazit für die Praxis

- Wir stehen an einem Wendepunkt in der globalen Akzeptanz von Telemedizin als Teil der medizinischen Standardversorgung. Dennoch gibt es bis zum heutigen Tag keinen verfügbaren Dienst, der eine zuverlässige automatisierte Diagnose über das Spektrum der gesamten Dermatologie liefert.
- In den nächsten Jahren muss die Forschung in der Teledermatologie Modelle für die Arbeit mit hochvariablen Smartphone-Bildern verfeinern, um Algorithmen-gestützte Ferndiagnosen im dermatologischen Alltag global zu ermöglichen.
- Die erfolgreiche Entwicklung entsprechender Technologien für die dermatologische Diagnose und ihre effektive Anwendung in der klinischen Praxis können nur mit der aktiven Beteiligung der dermatologischen Gemeinschaft erfolgen.
